# Revision of the genus *Vanenga* Schaus, 1928 (Lepidoptera, Mimallonoidea, Mimallonidae) with the description of a new species

**DOI:** 10.3897/zookeys.644.10705

**Published:** 2017-01-10

**Authors:** Ryan A. St. Laurent, Daniel Herbin

**Affiliations:** 1 McGuire Center for Lepidoptera and Biodiversity, Florida Museum of Natural History, University of Florida, 3215 Hull Road, Gainesville, FL 32611-2710 USA; 2 7, Le Clos de Lutché, F-31380 Garidech, France

**Keywords:** Argentina, Brazil, Paraguay, Uruguay, Vanenga mera, Vanenga mediorosea sp. n.

## Abstract

*Vanenga* Schaus, 1928, like many other Mimallonidae genera being revised in recent years, has not been studied since Schaus (1928) in his revision of the family. This currently monotypic genus is entirely restricted to South America, with no known representatives in Central or North America. Prior to this work, Schaus (1928) and subsequent lists of the family (Gaede 1931, Becker 1996) have mentioned the single species *Vanenga mera* (Dognin, 1924) described from the Brazilian Amazon (Pará state). In Schaus (1928) this species is listed as occurring in both Amazonia and southeastern Brazil.

In completing the present article, numerous “type” specimens have been discovered bearing three different manuscript names associated with the populations of southeastern Brazil and adjacent areas. Despite the fact that these names were written on various labels, the absence of any published descriptions results in them being unavailable (ICZN 1999). Therefore, this distinct southern South American species is now officially recognized and formally described, as well as providing a much more thorough distribution for both *Vanenga* species, including many new records for *Vanenga mera*.

## Introduction

Dissections were performed as in Lafontaine (2004). Morphological, including genitalia, terminology follows Kristensen (2003). Not all genitalia were prepared on slides to allow for three-dimensional analysis of the complex male genitalia. Genitalia and abdomens, when not slide mounted, are preserved in glycerol filled microvials.

Specimens from the following collections were examined:

ADW
 Coll. of Andrew D. Warren, Castle Rock, Colorado, USA 


CDH
 Coll. of Daniel Herbin, Garidech, France 


CGCM
 Coll. of Carlos G. C. Mielke, Curitiba, Paraná, Brazil 


CNC
 Canadian National Collection of Insects, Arachnids and Nematodes, Ottawa, Ontario, Canada 


CPAC
 Coleção Embrapa Cerrados, Planaltina, Distrito Federal, Brazil 


CUIC
Cornell University Insect Collection, Ithaca, New York, USA 


DZUP
 Collection of Pe. Jesus S. Moure, Departamento de Zoologia, Universidade Federal do Paraná, Curitiba, Paraná, Brazil 


MWM
Museum Witt, Munich, Germany 


NHMUK
Natural History Museum, London, U.K. 


USNM
National Museum of Natural History [formerly United States National Museum], Washington D.C., USA 


The symbol ‡ will be used to represent unavailable names in the text (Fletcher and Nye 1982).

Figures were manipulated with Adobe Photoshop CS4 (Adobe 2008). Male genitalia are figured in natural color with CS4 “auto color” used to improve white backgrounds. All geographical coordinates are approximate, and are based on the localities provided on specimen labels. GPS data were acquired with Google Earth.

## Results and discussion

### Vanenga

Taxon classificationAnimaliaLepidopteraMimallonidae

Schaus, 1928: 664

#### Type species.

*Perophora mera* Dognin, 1924; Schaus 1928: 664, by original designation.

#### Diagnosis.

The *Vanenga* species are small, relatively plain mimallonids, but can be recognized by the short triangular forewings, straight, preapical or apical postmedial line, pale tan-orange ground color with varying degrees of pink coloration throughout the antemedial and medial areas, which may strongly contrast against the darker orange-brown submarginal area. The male genitalia define the genus by being rather simple with triangular valves, very long and narrow uncus, heavily sclerotized lobe-like gnathos projections, and a short, thick phallus with accessory spiny dorsal projections emanating from the juxta-phallus complex. These juxtal projections may be either long and narrow or short. The male genitalia are reminiscent of some species of *Lacosoma* Grote, 1864, as mentioned by Schaus (1928), but can be distinguished by the longer, narrower uncus and phallic structure. The female genitalia are unique in the absence of a clear lamella antevaginalis and by possessing a very broad ductus bursae, the papillae anales are also quite narrow and elongated, a combination of characters so far seen in no other female Mimallonidae.

#### Description.

**Male.** *Head*: Varying shades of orange, eyes very large, more than two thirds area of head; antenna coloration as for head, bipectinate to tip, though distal fifth of pectinations much reduced in size; labial palpus highly reduced, three segmented, palpus not extending beyond frons. *Thorax*: Straw colored or with pale pink scales, darker brown-orange scales present on prothoracic collar. *Legs*: Coloration as for thorax, but usually darker orange with rosy scales, vestiture fine. Tibial spurs short, curved, naked or dorsally clothed in scales. *Forewing dorsum*: Forewing length: 10–17 mm, wingspan: 23.0–32.5 mm. Rather short, not elongated, triangular, apically somewhat rounded or more angled. Ground color pale tan-orange, with varying degree of pinkish hue throughout, especially antemedial and medially, overall lightly speckled by dark brown petiolate scales, though may be nearly absent. Antemedial line absent or as faint brown wavy mark. Postmedial line nearly straight, preapical or apical, brown, fading after passing Rs3 or Rs4. Antemedial and medial areas concolorous, either orange-tan or pale pink. Submarginal area always darker than medial area, usually orange-brown with diffuse gray coloration along wing margin, petiolate scales more abundant submarginally. Discal spot may be absent, when present as small dark brown spot. Fringe orange-tan or with light pink hue. *Forewing ventrum*: Similar to dorsum but coloration of medial and submarginal areas more similar, more uniformly orange, becoming gray distally, usually also pink nearer to thorax. Discal spot always present, usually larger than on dorsum (when present there), sometimes more elongated, narrower. Postmedial line somewhat wavy, not as straight as on dorsum, preapical. Petiolate scales more abundant, widely dispersed. *Hindwing dorsum*: Rounded, coloration and patterning as for forewing dorsum, antemedial line absent, postmedial line straight or curved outward, submarginal area broader than on forewing dorsum. *Hindwing ventrum*: Follows same pattern as forewing ventrum. Frenulum as single bristle. *Venation*: Typical of Mimallonidae, similar to *Lacosoma* Grote, 1864 but distal margin of discal cell more slanted. *Abdomen*: Concolorous with thorax, ventrally with pair of darker gray lines of scales, distal tip of abdomen with tuft of black scales, usually upturned in well-preserved specimens. *Genitalia*: Simple; vinculum ovoid, ventrally with lightly sclerotized plate attached to VIII. Uncus simple, acutely triangular but very elongated and narrow, laterally smoothly curving or slightly indented mesally. Gnathos formed by two rather short, unfused arms, as either ovoid, flattened lobe with triangular tooth emanating from center or as more heavily sclerotized downwardly angled cylindrical protuberance. Valves triangular, somewhat angled distally. Valves with baseo-mesal indentation which usually bears patch of thick, heavy setae; base of valves extend past vinculum inward into body cavity as narrow singular or bifurcated extension loosely connected to diaphragm and juxta. Juxta fused to phallus, encircling it as oddly shaped saucer with dorsal spined projections superior to phallus, projections either short and heavily spined or more elongated, curved, and less heavily spined. Phallus short, cylindrical, not much longer than width of juxtal saucer. Vesica bag-like. **Female.** *Head*: As in male but antennae much smaller overall, pectination particularly shorter. *Thorax*: As in male though brown scales along prothoracic collar may be darker. *Legs*: As in male, but tibial spurs shorter. *Forewing dorsum*: Forewing length: 12–15 mm, wingspan: 25–31 mm. Sexual dimorphism reduced, male and female very similar. Maculation similar to male but wing broader, convex mesally, submarginal area broader, apex more sharply acute. Coloration generally more diffuse between antemedial, medial, and submarginal areas, less distinctly bicolored. Submarginal area grayer or pinker overall than medial area relative to males. Postmedial line usually more bowed outward, though may be just as straight as in males. *Forewing ventrum*: Similar to dorsum but darker or paler orange. *Hindwing dorsum*: Coloration and patterning as for forewing dorsum, similar to males but differing in same characters as forewing dorsum. *Hindwing ventrum*: Follows same pattern as forewing ventrum. Frenulum reduced, as multiple bristles. *Abdomen*: Concolorous with thorax, ventrally darker, distal tip with small tuft of elongated scales. *Genitalia*: Small overall, most characters somewhat atrophied, VIII as weakly sclerotized plate, posteriorly curved, laterally VIII more heavily sclerotized. Apophyses anteriores less than half-length of apophyses posteriores or absent, apophyses posteriores elongate, narrower than apophyses anteriories (when present). Lamella antevaginalis membranous. Ductus bursae broad, wrinkled, bag-like. Corpus bursae narrow, elongated, nearly twice length of VIII–IX. Papillae anales somewhat flattened ventrally, elongated and narrow.

#### Remarks.

The genus *Vanenga* seems to share a close affinity with *Lacosoma* considering the male genitalia (Schaus 1928, St Laurent and Herbin pers. obs.) and small size of these moths overall. However, the female genitalia and external coloration, pattering, and wing shape are all quite distinct from *Lacosoma*. Compare our Figs 10–14 to male/female *Lacosoma* genitalia figured in Herbin and Mielke (2014), Herbin and Monzón (2015), and Herbin (2016).

### Vanenga mera

Taxon classificationAnimaliaLepidopteraMimallonidae

(Dognin, 1924)

**Figures 1–8.**
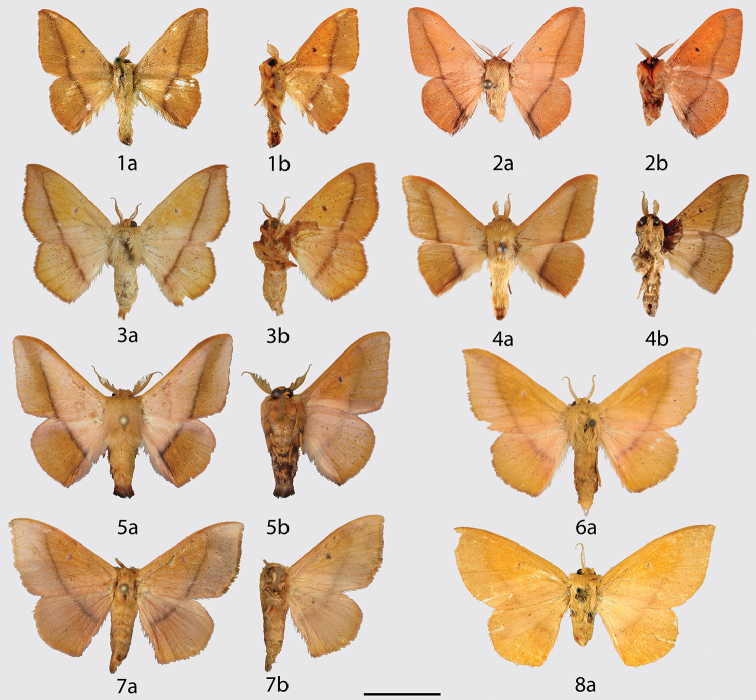
*Vanenga* adults, **a** dorsal **b** ventral. **1** *Vanenga mera* holotype ♂, Brazil, Pará, Óbidos (USNM) **2** *Vanenga mera* ♂, French Guiana, Route de Mana PK2 (CDH) **3** *Vanenga mera* ♀, Guyana, Tumatumari, Rio Potaro (CUIC) **4** *Vanenga mediorosea* holotype ♂, Brazil, Santa Catarina, Jaraguá do Sul (CUIC) **5** *Vanenga mediorosea* paratype ♂, Brazil, São Paulo, São José do Barreiro, Bocaina, 1539 m (CGCM) **6** *Vanenga mediorosea* paratype ♀, Argentina, Misiones, Iguazu (MWM) **7** *Vanenga mediorosea* paratype ♀, Brazil, Santa Catarina, São Bento do Sul, Rio Natal, 550 m (CGCM) **8** *Vanenga mediorosea* ♀, “type” of *Macessoga flavirosa*‡ Jones (manuscript name), Brazil, Paraná, Castro (NHMUK). Scale bar: 1 cm.

Figs 1–3 , 10 , 13 , 15

Perophora mera Dognin, 1924: 31
Vanenga mera ; Schaus 1928: 664, fig. ♂ 86g [incorrectly labeled as “asea” on plate, while the species Lacosoma asea Schaus, 1928 is labeled as “mera”]
Vanenga mera ; Gaede 1931
Vanenga mera ; Becker 1996

#### Type material.

**Holotype**, ♂. **BRAZIL: Pará**: Obidos, Amazones, Brésil [Óbidos, Pará]/ Dognin Collection/ Spec fig/ *Perophora mera* Type ♂, Dognin/ Type No. 29702 U.S.N.M./ USNM-Mimal: 1100/ (USNM). [examined].

#### Additional specimens examined.

(4 ♂, 1 ♀ total) **FRENCH GUIANA**: 1 ♂, Route de Mana PK2: 27.VII.2001, M. Laguerre, genitalia prep. D. Herbin ref. H 1118, Bc-Her 2945 (CDH). **GUYANA**: 1 ♂, Tumatumari: I.1908, S.M. Klages, Rothschild Bequest B.M. 1939–1, St Laurent diss.: 7-7-16:2 (NHMUK). 1 ♀, Tumatumari, Rio Potaro, St Laurent diss.: 5-17-16:5 (CUIC). **BRAZIL: Amazonas**: 1 ♂, São Paulo de Olivença, Rio Solimões: 22.II.1930, H. S. Parish, Cornell U. Lot 672, Sub 386 [abdomen missing, no genitalia preparation] (CUIC). **Roraima**: 1 ♂, Ilha de Maracá, Alto Alegre: 26.XI–2.XII.1987, Mielke & Casagrande (DZUP).

#### Diagnosis.

*Vanenga mera* can be distinguished from the following species in both sexes. Usually *Vanenga mera* is smaller, and always bears a distinct discal mark which is often absent in *Vanenga mediorosea* sp. n. In both sexes, the postmedial line terminates apically in *Vanenga mera* but is clearly preapical in *Vanenga mediorosea* sp. n. In males, the forewings are stouter and less sharply angled apically, and the ground color is more tan-orange to fawn, with very little if any pink coloration. Ventrally, the forewing postmedial line is wavier in *Vanenga mera*. The female is much lighter colored dorsally than the female of *Vanenga mediorosea* sp. n., with an almost yellow ground color and a pinkish hue submarginally, unlike the brown to orange-brown females of *Vanenga mediorosea* sp. n. which are usually more grayish brown submarginally. Genitalia are also useful in differentiating these two species. In *Vanenga mera* the uncus is not indented when viewed laterally and the gnathos consists of flattened, ovoid lobes with a single tooth mesally, rather than the heavily sclerotized, thumb-like projections of *Vanenga mediorosea* sp. n. Finally, the valves are broader, and the dorsal projections of the phallus-juxta complex are elongated, curved, and only weakly spined, not short, stout, and heavily spined as in *Vanenga mediorosea* sp. n. The female genitalia can be differentiated from those of *Vanenga mediorosea* sp. n. by the sclerotized ring of VIII, which is incomplete in *Vanenga mera* and complete in *Vanenga mediorosea* sp. n. Furthermore, the apophyses are longer in *Vanenga mera*, with the apophyses anteriores actually being absent in *Vanenga mediorosea* sp. n.

#### Description.

**Male.** *Head*: As for genus but darker orange to almost red-orange. *Thorax*: As for genus but pale pink scales absent. *Legs*: Coloration as for thorax, but with darker orange scales, especially on tibia and tarsus, similar in coloration to that of head. Tibial spurs short, curved, naked or dorsally clothed in scales. *Forewing dorsum*: Forewing length: 11–13 mm, avg.: 12 mm, wingspan: 24–25 mm, avg.: 24.5 mm [26 mm in Dognin (1924)], n = 2. Short, stout, subtriangular, apically rounded, margin nearly straight to slightly convex. Ground color pale tan-orange, overall lightly speckled by dark brown petiolate scales, particularly submarginally. Antemedial line absent or as faint brown wavy mark. Postmedial line nearly straight, apical, brown. Antemedial and medial areas concolorous, pale orange-tan. Submarginal area darker orange-brown compared to medial area, usually orange-brown with pale diffuse gray coloration near apex. Discal spot always present as small dark gray-brown spot. Fringe orange-tan. *Forewing ventrum*: Similar to dorsum but darker, more uniformly orange, becoming gray distally. Discal spot always present, usually larger, darker than on dorsum. Postmedial line preapical, not as straight as on dorsum. Petiolate scales more abundant, widely dispersed. *Hindwing dorsum*: Rounded, coloration and patterning as for forewing dorsum, antemedial line absent, postmedial line mostly straight, submarginal area broader than on forewing dorsum. *Hindwing ventrum*: Follows same pattern as forewing ventrum. *Abdomen*: As for genus. *Genitalia*: (Fig. 10) n = 2. As for genus but uncus laterally smoothly curving or nearly straight, not indented mesally. Gnathos as pair of ovoid, flattened lobes with sharp tooth emanating from center of lobe. Valves triangular, somewhat angled distally, relatively broad. Valves with baseo-mesal indentation bearing thick patch of heavy setae; base of valves barely extend past vinculum inward into body cavity as narrow, triangular extension loosely connected to diaphragm and juxta. Juxta fused to phallus, encircling it as oddly shaped saucer with two pairs of elongate, curved, weakly spined projections superior to phallus, one pair longer than the other. Phallus short, cylindrical, not much longer than juxtal extensions. **Female.** *Head*: As in male but broader, antennae much smaller overall, pectinations particularly shorter. *Thorax*: As in male. *Legs*: As in male, but tibial spurs shorter. *Forewing dorsum*: Forewing length: 15 mm, wingspan: 27 mm, n = 1. Similar to male but broader, margin convex mesally, submarginal area broader, apex more acute. Coloration lighter, lemon yellow, more diffuse between antemedial, medial, and submarginal areas, less distinctly bicolored. Submarginal area pinker than medial area. *Forewing ventrum*: Similar to dorsum but generally darker orange. *Hindwing dorsum*: Coloration and pattern as for forewing dorsum. *Hindwing ventrum*: Follows same pattern as forewing ventrum but with more pinkish-gray hue. *Abdomen*: Concolorous with thorax, ventrally darker, distal tip with small tuft of elongated scales. *Genitalia*: (Fig. 13) n = 1. Small overall, most characters atrophied, VIII as weakly sclerotized plate, posteriorly curved, laterally VIII more heavily sclerotized. Apophyses anteriores less than half length of apophyses posteriores but robust, terminating in flat tip, apophyses posteriores elongate, curving outward, narrower than apophyses anteriores. Lamellae absent, replaced by broad membranous area. Ductus bursae broad, bag-like. Corpus bursae narrow, elongated, nearly twice length of VIII–IX. Papillae anales flattened ventrally, elongated and narrow.

**Figure 9.**
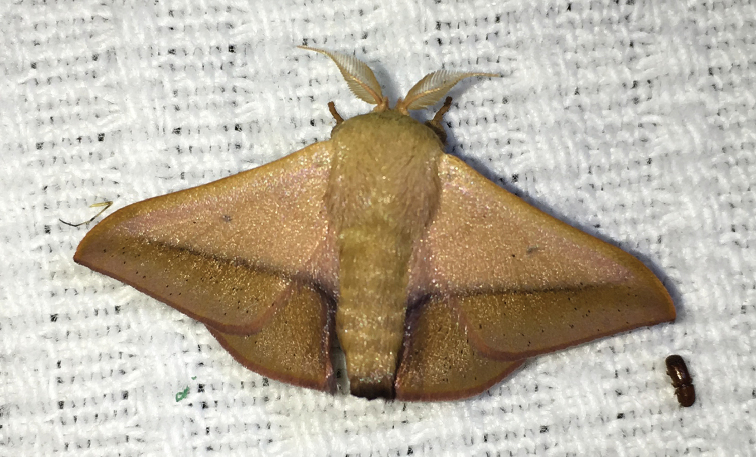
*Vanenga mediorosea* ♂, Brazil, Rio Grande do Sul, Santa Maria, 15.XII.2015, at MV light, photo R. St Laurent (specimen not collected).

**Figures 10–12.**
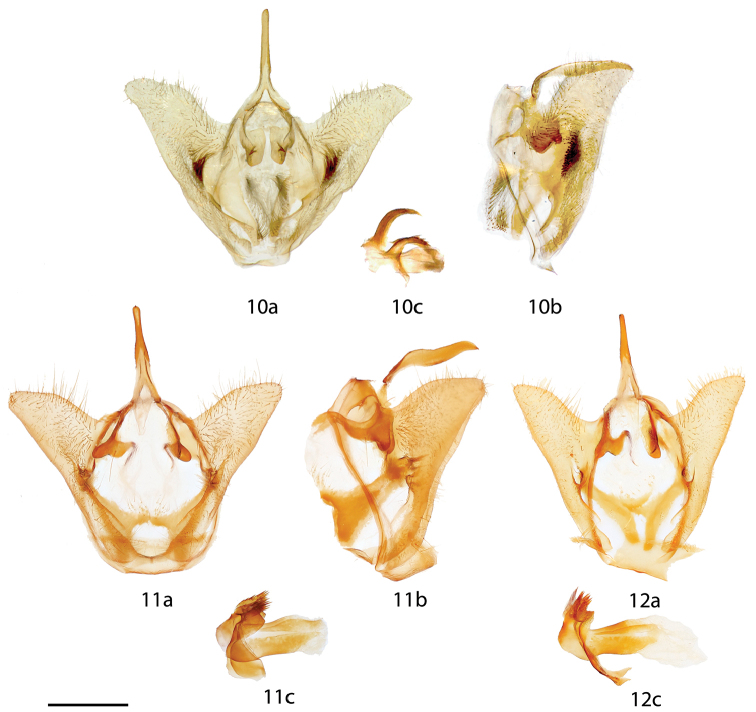
*Vanenga* male genitalia, **a** ventral **b** lateral **c** phallus. **10** *Vanenga mera*, Guyana, Tumatumari, St Laurent diss.: 7-7-16:2 (NHMUK) **11** *Vanenga mediorosea* holotype, Brazil, Santa Catarina, Jaraguá do Sul, St Laurent diss.: 5-17-16:1 (CUIC) **12** *Vanenga mediorosea* paratype, Brazil, Rio Grande do Sul, Guarani das Missões, St Laurent diss.: 2-26-16:5 (CUIC). Scale bar: 1 mm.

**Figures 13, 14.**
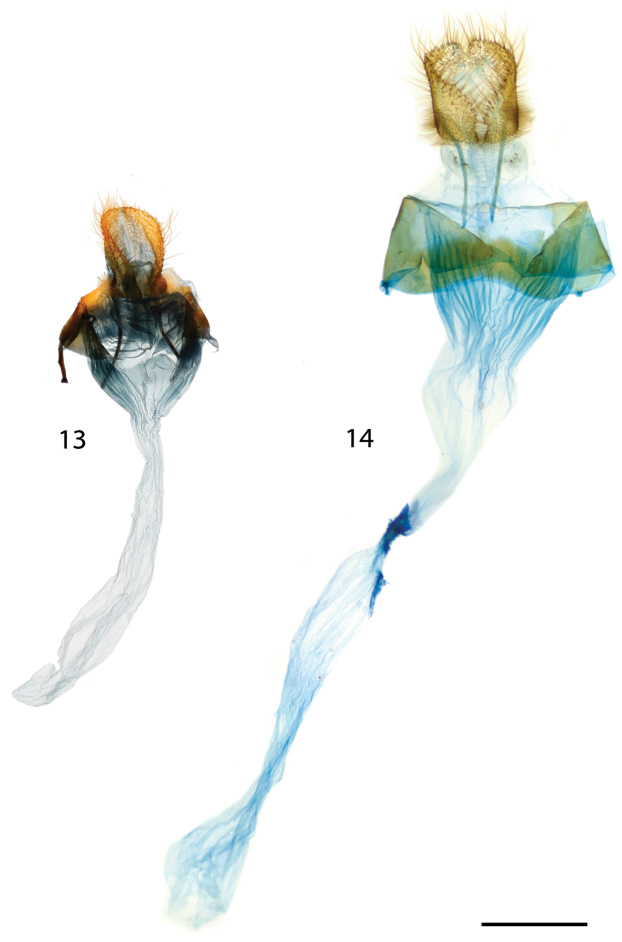
*Vanenga* female genitalia. **13** *Vanenga mera* Guyana, Tumatumari, Rio Potaro, St Laurent diss.: 5-17-16:5 (CUIC) **14** *Vanenga mediorosea* paratype, Argentina, Missiones, Iguazu, genitalia prep. No. 29.236 (MWM). Scale bar: 1 mm.

#### Distribution

**Figure 15.**
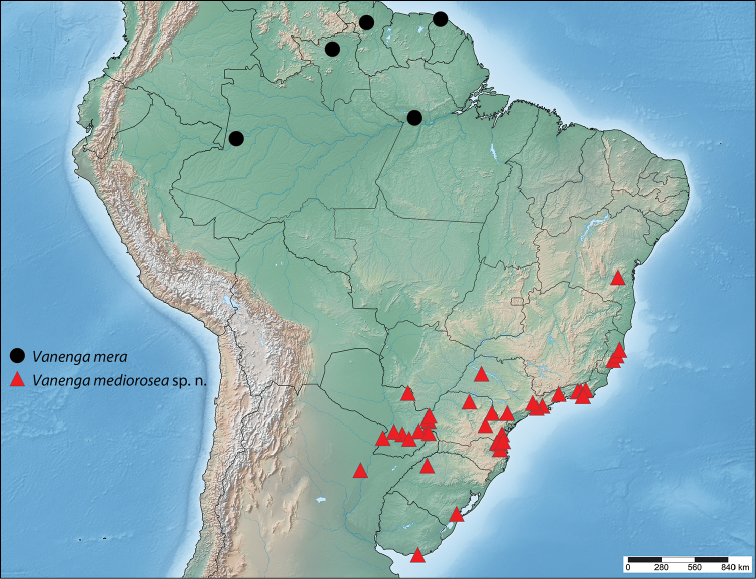
Known distribution of *Vanenga*.

**(Fig. 15).** This species has an Amazonian distribution, and has so far been collected in Guyana, French Guiana, and the Brazilian states of Amazonas, Pará, and Roraima.

#### Remarks.

Until the present work, all *Vanenga* specimens have been treated as *Vanenga mera* in the literature and various worldwide collections, but external and genital morphology, as well as biogeography, clearly separates the genus into two well-distinct species, with the name *Vanenga mera* only being applicable to the rarely collected Amazonian species. In Schaus’s (1928) treatment of Mimallonidae, he considered all populations to be this one species; hence his records for Southeastern Brazil and Paraguay can be attributed to the following new species, now formally described below.

### Vanenga mediorosea sp. n.

Taxon classificationAnimaliaLepidopteraMimallonidae

http://zoobank.org/8E8A6963-2C55-4E40-8B71-78AA20BA3DEA

Figs 4–8 , 9 , 11 , 12 , 14 , 15

Vanenga mera ; Schaus 1928, in part

#### Type material.

**Holotype**, ♂. **BRAZIL: Santa Catarina**: Jaragua [Jaraguá do Sul], Santa Catarina, Brazil, 5 Dec 1935, Fritz Hoffman/ *Vanenga mera* Dognin [illegible]?/ St Laurent diss.: 5-17-16:1/ HOLOTYPE male *Vanenga mediorosea* St Laurent and Herbin, 2017 [handwritten red label]/ (CUIC).

**Paratypes.** (103 ♂, 8 ♀ total) **BRAZIL: Bahia**: 1 ♂, Jequié, 13°56'S, 40°11'W: III.2012, H. Thöny leg., genitalia prep. No. 29.240 (MWM). **Espírito Santo**: 7 ♂, Santa Leopoldina, Dorf Tirol, 24°75'S, 40°50'W [coordinates may be incorrect], 700 m: 22–31.X.1996 (2 ♂), 8–20.XII.1996 (3 ♂), genitalia prep. No. 29.237, VI.1998 (1 ♂), X.1999 (1 ♂), H. Thöny leg. (MWM). 1 ♂, Santa Leopoldina, Dorf Tirol, 20°10'S, 40°33'W, 700 m: XI.2000, H. Thöny leg. (MWM). 1 ♂, Aracruz: 12.III.1993, João B. Silva [leg.], Coleção Embrapa-CPAC No. 20903 (CPAC). 8 ♂, Linhares, 40 m: 20–29.II.1992 (2 ♂), 5–9.IV.1992 (5 ♂), 25–30.I.1998 (1 ♂), V.O. Becker col., Col. Becker 80934, 82021, 113494, USNM-Mimal: 2059, 2171–2175, 2185, 2186 (USNM). 2 ♂, No additional locality data: USNM-Mimal: 1683, 2539 (USNM). **Rio de Janeiro**: 1 ♂, 1 ♀, Petrópolis: 4.XII.1928, 24.IV.1960, Gagarin leg., ex. col. Gagarin (DZUP). 1 ♂, Angra-Jussaral: 28.XI.1935, No. 19.200 (DZUP). 1 ♂, Barreira, Teresópolis, 400 m: 26–29.IV.1957, Pearson H. G., No. 19.199 (DZUP). 1 ♀, Indepêndencia, Petrópolis, 900 m: 16.X.1934, Gagarin leg., ex. col. Gagarin (DZUP). 1 ♂, Reserva Ecológica de Guapiaçu, Cachoeiras de Macacu: 25.I.2011, Tangerini Col., Ex. col. Nirton Tangerini (DZUP). 1 ♂, Boca do Mato, Cachoeira de Macacu: 11–20.X.1996, Tangerini leg. (MWM). 7 ♂, Maricá, 5 m: 12–15.I.1985 (5 ♂), 11.X.1995 (2 ♂), V.O. Becker col., Col. Becker 54454, 65200, USNM-Mimal: 2169, 2170, 2177–2181, St Laurent diss.: 8-22-16:5 (USNM). **São Paulo**: 3 ♂, Est. Biol. Boracéia, nr. Salesópolis, 850 m: 13.III.1972 (1 ♂), 14.III.1972 (2 ♂), St Laurent diss.: 5-17-16:2, E.G., I. & E.A. Munroe (CNC). 1 ♂, Salesópolis, Boracea [Boracéia], 850 m: 14–18.II.1950, Trav. Trav. Filho, Pearson, & Rabello coll., Brit. Mus. 1962–112 (NHMUK). 2 ♂, Boracéa [Boracéia], Salesópolis: 8–14.II.1959, Travassos, Kloss, & Pearson leg., HRP 2090, 2091, 2094, *Vanenga mera* Dognin Pearson det., USNM-Mimal: 2182, 2183 (USNM). 3 ♂, Guapiara, Paivinha, 800 m: 2–5.V.2005, 18–21.XII.2005, 3–6.IV.2007, C. Mielke leg., Col. C. Mielke 28.580, 30.041, 32.043 (CGCM). 2 ♂, São José do Barreiro, Bocaina, 22°43'37"S, 44°37'57"W, 1539 m: 2–6.I.2016, C. Mielke leg., Col. C. Mielke 31.330, 31.335, C. Mielke gen. prep. 31.330 (CGCM). 8 ♂, Alto da Serra [Paranapiacaba]: XI.1922, XII.1922, III.1925, II.1926, IV.1926, R. Spitz, Rothschild Bequest, B.M. 1939–1 (NHMUK). 1 ♂, Cantareira: Coll. R. Spitz, Brit. Mus. 1962–112 (NHMUK). 1 ♂, Ypiranga [Ipiranga]: IV.1924, R. Spitz, Rothschild Bequest, BM 1939–1 (NHMUK). 1 ♀, Araçatuba, 450 m: 2.IV.1913, E.D. Jones, E.D. Jones Coll., Brit. Mus. 1919–295, St Laurent diss.: 7-7-16:3 (NHMUK). **Paraná**: 1 ♂, Ponta Grossa: I.1956, Coleção F. Justus Jor, at light (DZUP). 1 ♂, Foz do Iguaçu, 200 m: 16.II.1969, Moure & Mielke (DZUP). 1 ♂, Rolândia: XII.1952, Maller col., Brit. Mus. 1962–112 (NHMUK). 10 ♂, Curitiba, Serra do Mar, Estrada de Castelhanos, 500 m: 30.XI.1997 (2 ♂), III.1998 (3 ♂), IV.1998 (5 ♂), H. Thöny leg. (MWM). 1 ♂, Jaguariaíva, Parque Estadual do Cerrado 24°10'4.98"S, 49°39'59.35"W: 28.II.2015, Andrew D. Warren leg., MV light (ADW). **Santa Catarina**: 1 ♂, Jaragua [Jaraguá do Sul]: 5.XII.1935, Fritz Hoffmann (CUIC). 2 ♂, Blumenau: X, Br. Pohl, Cornell U. Lot 819, Sub 322, “Paratype” [blue label], *Cicinnus roseatincta*‡ Schaus No. 697 Paratype [manuscript name], St Laurent diss.: 5-17-16:3 (CUIC). 1 ♂, Blumenau: “671,” USNM-Mimal: 2538 (USNM). 1 ♀, São Bento do Sul, Rio Natal, 550 m: XI.2013, A. Rank leg., Col. C. Mielke 28.007 (CGCM). 2 ♂, São Bento do Sul, Serra Rio Natal, 850 m: VII.1998, XI.1998, H. Thöny leg., genitalia prep. No. 29.238, 29.239 (MWM). 3 ♂, No additional locality data: F. Hoffman, USNM-Mimal: 2534, 2535, 2537, specimen 2537 with label “Saturniidae ?” (USNM). 1 ♂, Hansa Humbolt [Corupá]: “10,” USNM-Mimal: 2540, St Laurent diss.: 8-22-16:4 (USNM). 1 ♂, Joinville, 500 m: 3.I.1989, V.O. Becker [leg.], Col. Becker 60597, USNM-Mimal: 2176 (USNM). **Rio Grande do Sul**: 5 ♂, Pelotas: 5.IV.1954, 27.III.1959, no date, C.M. Biezanko, B.M. 1954–395, 1961–209 (NHMUK); 18.III.1953, 15.IV.1953, C.M. de Biezanko, No. 753, St Laurent diss.: 5-17-16:4 (CUIC). 2 ♂, Guarani [das Missões]: 3.III.1932, C.M. de Biezanko, No. 753, St Laurent diss.: 2-26-16:5 (CUIC). **PARAGUAY: Guairá**: 1 ♀, Villarica: XI.1927, F. Schade [leg.], Joicey Bequest, Brit. Mus. 1934–120 (NHMUK). 1 ♀, Villarica: 18.IV.1925, F. Schade Coll., Collection Wm Schaus, USNM-Mimal: 2541, [handwritten label in Schaus’s handwriting:] “*Cicinnus meroides*‡ [or *meroidea*‡] type Schaus mss” (USNM). **Caazapá**: 1 ♂, Cristal, San Juan de Nepomuceno: XII.1998 (MWM). **Alto Paraná**: 1 ♂, Reserva Biológica Limoy, 24°47'S, 54°26'W: 17–20.IV.1986, M. Pogue & M. Solis [leg.], USNM-Mimal: 2407 (USNM). 1 ♂, Limoy, 24°45'S, 54°27'W, 245 m: 01–05.XI.2009, U. Drechsel [leg.] (CDH). 1 ♂, 1 ♀, Estancia Dimas, 25°33'S, 55°13'W, 200 m: 24–26.III.2011 (1 ♂), 26–31.I.2012 (1 ♀), U. Drechsel [leg.] (CDH). **Canindeyú**: 4 ♂, Armisticio, 24°34'S, 54°32'W, 290 m: 26–30.XI.2009, U. Drechsel [leg.] (CDH). 1 ♂, Carapá, 24°22'S, 54°23'W, 240 m: 1–4.IV.2009, U. Drechsel [leg.] (CDH). **Ñeembucú**: 1 ♂, Zanjita, 26°03'S, 57°56'W, 50 m: 1–3.III.2013, U. Drechsel [leg.] (CDH). **Paraguarí**: 1 ♂, Mbatoví, 25°35'S, 57°05'W, 383 m: 17–18.IV.2014, U. Drechsel [leg.], genitalia prep. D. Herbin ref. H 1120, Bc-Her4967 (CDH). **Amabay**: 1 ♂, Parque nacional Cerro Corá, 22°39'S, 56°01'W: 7–10.IV.1986, M. Pogue & M. Solis [leg.], USNM-Mimal: 2757, St Laurent diss.: 8-22-16:6 (USNM). **ARGENTINA: Misiones**: 1 ♂, 1 ♀, Iguazu: 23–26.XI.1993, J.R., genitalia prep. no. 29.236 [♀] (MWM). **Santa Fe**: 4 ♂, Villa Ana, Ferrocarril Provincial de Santa Fe: III.1924, K.J. Hayward [leg.], Brit. Mus. 1924–203, [genitalia] vial NHMUK010402135, NHMUK010318285 (NHMUK). **URUGUAY: Maldonado**: 1 ♂, Piriápolis: 8.II.59 [interpretation of “8/11/59”], F. Penades [leg.] (NHMUK). – All paratypes with the following yellow label: PARATYPE male/female *Vanenga mediorosea* St Laurent and Herbin, 2017.

#### Additional specimens examined.

[not to be included in type series] (1 ♂, 1 ♀ total) **BRAZIL: Rio Grande do Sul**: 1 ♂, Pelotas: 18.III.1953, C.M. de Biezanko [leg.], No. 753 (ex. CUIC donated to CMNH). **Paraná**: 1 ♀, Castro, 950 m: E.D. Jones, E.D. Jones Coll. Brit. Mus. 1919–295, “*Perophora flavirosa*‡ Type ♀ D-Jones” [manuscript name], “*Macessoga flavirosa*‡ (Jones) type genit. pr. No 9, Mimallonidae” [genitalia prep. lost], NHMUK010354541 (NHMUK).

#### Photo of living specimen examined.

[not to be included in type series] **BRAZIL: Rio Grande do Sul**: 1 ♂, Santa Maria, -29.697441°, -53.920125°, 119 m: 15.XII.2015, R.A. St. Laurent & A.P.S. de Carvalho leg., at MV light (Fig. 9).

#### Diagnosis.

For characters differentiating this new species from the previous one, see the diagnosis of *Vanenga mera*.

#### Description.

**Male.** *Head*: As for genus, varying shades of orange fading to pale tan. *Thorax*: Straw colored, usually with pale pink scales, darker brown-orange scales may be present on prothoracic collar. *Legs*: Coloration as for thorax, but usually darker orange with rosy scales, vestiture fine, tibial spurs short, curved, naked or dorsally clothed in scales. *Forewing dorsum*: Forewing length: 10–17 mm, avg. 13.7 mm, wingspan: 23.0–32.5 mm, avg. 27.2 mm, n = 50. Triangular, apically angled, margin nearly straight though may be barely concave or convex. Ground color pale tan-orange to gray-salmon, with varying degree of pale to strong pink coloration antemedial and medially, overall lightly speckled by dark brown petiolate scales, though petiolate scales often absent medially. Antemedial line absent or as faint brown or gray wavy mark. Postmedial line preapical, faint, straight, dark brown to black, distally curved to costa, fading after passing Rs3 or Rs4. Antemedial and medial areas concolorous, pale pink. Submarginal area always darker than medial area, usually orange-brown with diffuse gray coloration along wing margin, petiolate scales more abundant. Discal spot usually absent, though sometimes present as small, dark brown or black spot. Fringe orange-tan with light pink hue. *Forewing ventrum*: Similar to dorsum but coloration of medial and submarginal area more similar, more uniformly darker orange, becoming gray distally, usually also pink nearer to thorax. Discal spot always present, oblong or circular. Postmedial line may be somewhat wavy or very faint. Petiolate scales more abundant, widely dispersed. *Hindwing dorsum*: Rounded, coloration and patterning as for forewing dorsum, antemedial line absent, postmedial line straight, submarginal area broader than on dorsum. *Hindwing ventrum*: Follows same pattern as forewing ventrum, but rosier pink overall than orange, especially antemedial and medially. *Abdomen*: As for genus. *Genitalia*: (Figs 11, 12) n = 15. As for genus but uncus indented mesally when viewed laterally. Gnathos as pair of heavily sclerotized, downward angled protuberances of variable thickness and length. Valves triangular, somewhat angled distally, relatively narrow, acute. Valves with baseo-mesal indentation usually bearing small patch of heavy setae; base of valves extend well past vinculum inward into body cavity as singular bifurcated extension loosely connected to diaphragm and juxta. Juxta fused to phallus, encircling it as oddly shaped saucer with two pairs of dorsal projections superior to phallus, projections short and heavily spined. Phallus short, cylindrical, not much longer than width of juxtal saucer. Vesica bag-like, small. **Female.** *Head*: As in male but antennae much smaller overall, pectinations particularly shorter. *Thorax*: As in male though may be darker brown overall. *Legs*: As in male, but tibial spurs shorter. *Forewing dorsum*: Forewing length: 12.0–14.5 mm, avg. 13.9 mm, wingspan: 26–31 mm, avg. 28.5, n = 4. Forewing broader than in male, more elongated, convex mesally, submarginal area broader, apex more acute. Coloration generally more diffuse between antemedial, medial, and submarginal areas, less distinctly bicolored. Overall darker orange-brown antemedial and medially, submarginal area lighter gray compared to medial area. Postmedial line usually more bowed outward than in male. *Forewing ventrum*: Similar to dorsum but generally paler orange. *Hindwing dorsum*: Coloration and pattern as for forewing dorsum. *Hindwing ventrum*: Follows same pattern as forewing ventrum. *Abdomen*: As for genus. *Genitalia*: (Fig. 14) n = 2. Small overall, most characters atrophied, VIII as moderately sclerotized ring, posteriorly curved, ventrally angled inward forming anteriorly directed angle with ostium at apex. Apophyses anteriores absent, apophyses posteriores elongate, but not much longer than length of extended IX. Sclerotized lamella antevaginalis absent, replaced by broad membranous area. Ductus bursae broad, bag-like. Corpus bursae narrow, elongated, more than twice length of VIII–IX. Papillae anales flattened ventrally, elongated and narrow.

#### Distribution

**(Fig. 15).** This new species is broadly distributed in the Brazilian Atlantic Forest in the states of Bahia south through Espírito Santo, Rio de Janeiro, São Paulo, Paraná, Santa Catarina, and Rio Grande do Sul. *Vanenga mediorosea* is also found in the Pampa biome of Rio Grande do Sul (Brazil) and Uruguay, and is found in neighboring Paraguay and Argentina in various habitats, particularly in inland forests (Alta Paraná) and Humid Chaco.

#### Etymology.

This species is named for the pink-flushed (*rosea* meaning pink, Latin) medial (*medio* Latin) area of the forewings.

#### Remarks.

As previously mentioned, *Vanenga mediorosea* is much more commonly collected than *Vanenga mera*, and thus the vast majority of *Vanenga* specimens are mislabeled as *Vanenga mera* in major collections. Furthermore, *Vanenga mediorosea* is often present in series in collections rather than singletons as is *Vanenga mera*.

In performing the research necessary for this revision, we have discovered “types” of *Vanenga* specimens bearing manuscript names, but which were apparently never described. In the NHMUK there is a single female specimen labeled as a type of *Perophora flavirosa*‡ Jones from Brazil, Paraná, (Fig. 8), curated together with other females of *Vanenga mediorosea*, and clearly this “type” belongs to the species that we describe herein. This specimen is also labeled as a “type” of *Macessoga flavirosa*‡ (Jones). However, we are unaware of any publication using this name in either combination by Jones from the period of time when he was describing Mimallonidae, and thus it is a manuscript name without an associated formal description. The genitalia preparation associated with this species is lost; therefore we omit it from the type series of *Vanenga mediorosea*.

Additionally, in the CUIC there are two specimens labeled as “paratypes” of *Cicinnus roseatincta*‡ Schaus, apparently from prior to 1928 since Schaus did not label these as *Vanenga* specimens (a genus he described for *Vanenga mera* in 1928). A holotype bearing this name has not been located. In Schaus (1928), he lists *Vanenga mera* as being present in Blumenau, Santa Catarina, Brazil, which incidentally, is the locality of these two “paratypes.” Therefore, we can infer that Schaus never formally described this species; otherwise the Brazilian specimens would not have been listed under *Vanenga mera* in his revision. Similarly, there is a female specimen at the USNM labeled as a “type” (in Schaus’s handwriting) of *Cicinnus meroides*‡ (or *meroidea*‡, the final letter is illegible, regardless in reference to “near *mera*”). This label bears the letters “mss” subsequently written in pencil over the name. Therefore, this seems to be yet a third manuscript name associated with this species, though again, never formally described for the same reasons as mentioned previously.

If any or all of these hereby-unavailable names is/are located in the literature, it would be necessary to treat *Vanenga mediorosea* as a synonym of the most senior name.

*Vanenga mediorosea* is rather consistent in coloration and markings across its range, though occasionally some specimens are darker brown-orange submarginally and more salmon colored medially. We also note some geographic variation in the length of the gnathos protuberance, which is shorter at the northern and southern extremities of the range (Bahia and Rio Grande do Sul respectively) than centrally along the distribution, such as those from Santa Catarina and São Paulo. There seems to also be some size variation depending on elevation, with larger specimens coming from higher elevations. Two male specimens (Brazil, São Paulo, near São José do Barreiro, 1539 m) from higher elevation than all other examined material are the largest of the examined specimens (Fig. 5). Additional specimens from Salesópolis (also São Paulo state) are also quite large in comparison with lower elevation material from coastal Brazil (such as Rio de Janeiro, Maracá and Espírito Santo, Linhares for example) and Paraguay. Genitalia of specimens from various elevations however, are consistent. It is interesting to note that the higher elevation specimens are actually larger than many from lower elevations.

## Supplementary Material

XML Treatment for Vanenga

XML Treatment for Vanenga mera

XML Treatment for Vanenga mediorosea
